# Effect of Ethanol, Sulfur Dioxide and Glucose on the Growth of Wine Spoilage Yeasts Using Response Surface Methodology

**DOI:** 10.1371/journal.pone.0128702

**Published:** 2015-06-24

**Authors:** Mahesh Chandra, Inês Oro, Suzana Ferreira-Dias, Manuel Malfeito-Ferreira

**Affiliations:** 1 Laboratório de Microbiologia, Linking Landscape, Environment, Agriculture and Food (LEAF), Instituto Superior de Agronomia, University of Lisbon, Tapada da Ajuda, 1349–017 Lisboa, Portugal; 2 Centro de Engenharia dos Biossistemas (CEER), Instituto Superior de Agronomia, University of Lisbon, Tapada da Ajuda, 1349–017 Lisboa, Portugal; University Paris South, FRANCE

## Abstract

Response surface methodology (RSM) was used to study the effect of three factors, sulfur dioxide, ethanol and glucose, on the growth of wine spoilage yeast species, *Zygosaccharomyces bailii*, *Schizosaccharomyces pombe*, *Saccharomycodes ludwigii* and *Saccharomyces cerevisiae*. Seventeen central composite rotatable design (CCRD) trials were designed for each test yeast using realistic concentrations of the factors (variables) in premium red wine. Polynomial regression equations were fitted to experimental data points, and the growth inhibitory conditions of these three variables were determined. The overall results showed *Sa*. *ludwigii* as the most resistant species growing under high ethanol/free sulfur dioxide concentrations, i.e., 15% (v/v)/20 mg L^-1^, 14% (v/v)/32 mg L^-1^ and 12.5% (v/v)/40 mg L^-1^, whereas other yeasts did not survive under the same levels of ethanol/free sulfur dioxide concentrations. The inhibitory effect of ethanol was primarily observed during longer incubation periods, compared with sulfur dioxide, which showed an immediate effect. In some CCRD trials, *Sa*. *ludwigii* and *S*. *cerevisiae* showed growth recovery after a short death period under the exposure of 20–32 mg L^-1^ sulfur dioxide in the presence of 11% (v/v) or more ethanol. However, *Sc*. *pombe* and *Z*. *bailii* did not show such growth recovery under similar conditions. Up to 10 g L^-1^ of glucose did not prevent cell death under the sulfur dioxide or ethanol stress. This observation demonstrates that the sugar levels commonly used in wine to sweeten the mouthfeel do not increase wine susceptibility to spoilage yeasts, contrary to the anecdotal evidence.

## Introduction

Yeasts play an important role in winemaking, favorably contributing to the quality and desirable properties of the wine. Occasionally, under uncontrolled conditions, the activities of yeast become detrimental to the wine quality. The most dangerous wine spoilage yeasts belong to the species *Zygosaccharomyces bailii* (*Z*. *bailii*), *Schizosaccharomyces pombe* (*Sc*. *pombe*), *Saccharomycodes ludwigii* (*Sa*. *ludwigii*) and *Brettanomyces bruxellensis* (*B*. *bruxellensis*), and even the fermenting species *Saccharomyces cerevisiae* (*S*. *cerevisiae*) might have undesirable properties [1]. The involvement of these yeasts in wine must be controlled to avoid any negative impact on the organoleptic quality of the final product [2].

Many studies have attempted to characterize the spoilage potential of wine related yeasts and assess the minimum acceptable number of yeasts in wine. Wine constituents, such as ethanol, sugar and sulfur dioxide, are the major factors influencing susceptibility to yeast growth [3, 4]. The fermentable sugars (glucose and fructose), present in sweet wines can act as energy and carbon sources for the growth of spoilage yeast, thereby increasing the hazard of wine spoilage [3]. Thus, the practice of adding sugar near the recognition threshold for sweetness (about 5.0 g L^-1^) to smoothen the mouthfeel with making its taste off-dry increases the concern of yeast spoilage among winemakers [5]. Therefore, yeast spoilage in low-sugar wines deserves much attention.

The response surface methodology (RSM) is a useful tool used for the estimation of the combined effects of different environmental variables on the growth of yeast [6, 7]. This methodology has been widely used in predictive microbiology as a secondary polynomial model to predict the microorganism response as a function of environmental changes to determine the interactions among these factors [8]. In a recent study, we used this methodology to demonstrate the interactions of ethanol, sulfur dioxide and residual glucose on the growth of *B*. *bruxellensis* in red wines [9]. In the same study, we observed the peculiar effect of glucose on *B*. *bruxellensis*. Despite being a suitable substrate for the growth of *B*. *bruxellensis*, low glucose concentrations up to 10 g L^-1^ did not support the growth of stressed cells. In contrast, high glucose levels increased the susceptibility of yeast to ethanol-mediated death. Therefore, it would be interesting to determine whether other dangerous wine spoilage species behave similarly. The aim of the present study was to characterize the effects of glucose, ethanol and sulfur dioxide and examine the influence of the interactions among these factors on the growth of a select group of wine spoilage species using a RSM approach.

## Materials and Methods

### Yeast strains and maintenance

The strains *Z*. *bailii* ISA 2270 (isolated from red wine), *Sa*. *ludwigii* ISA 1083 (isolated from sweet white wine), *Sc*. *pombe* ISA 1190 (strain CECT 1375 obtained from the Coleccion Española de Cultivos Tipo) and *S*. *cerevisiae* 1000 (commercial starter Fermivin) were maintained in slants of GYP medium containing 20 g L^-1^ glucose (Merck, Darmstadt, Germany), 5 g L^-1^ yeast extract (Difco Laboratories, Detroit, USA), and 10 g L^-1^ peptone (Difco Laboratories, Detroit, USA), pH 6.0, at 4 °C.

### Culture conditions

The yeast inocula were prepared through cultivation in 100 mL of Yeast Nitrogen Base (YNB, Difco) (6.7 g L^-1^) medium supplemented with glucose (20 g L^-1^) and ethanol (10%, v/v). The pH of medium was adjusted to 3.5 ± 0.01 using 0.1 M NaOH or 0.1 N HCl, followed by sterilization using membrane filtration (0.22 μm pore size). The culture was incubated at 25 °C with orbital shaking (120 rpm). Yeast growth was measured as the absorbance at 640 nm. When approximately 0.5 units were obtained, the wines were inoculated to generate an initial population of approximately 10^5^ cells mL^-1^ of each test yeast species. The wines were subsequently incubated at 25°C in 100 mL Erlenmeyer flasks capped with rubber plugs with inserted hypodermic needles and a minimal amount of headspace to minimize evaporation and to avoid oxidation of wine. During incubation, the wine samples were decimally diluted, and cellular culturability was determined after surface plating 0.025 mL of the wine sample onto GYP medium, in duplicate, followed by incubation at 25°C for up to 7 days.

Experimental wines were obtained from a blend of several commercial red wines without residual sugar (< 2 g L^-1^). The sulfur dioxide concentration was adjusted using potassium metabisulfite or removed using acetaldehyde [10]. The ethanol content was adjusted to different concentrations using a 5 g L^-1^ of tartaric acid solution (Merck) or 99% pure ethanol (Merck) [11]. The pH value was adjusted to 3.5 using concentrated NaOH (Merck) or HCl (Merck). The final wine blends were sterilized through membrane filtration (0.22 μm pore size and 47 mm diameter, Millipore).

### Chemical analysis

#### Conventional analysis

The ethanol content, pH, density, total and volatile acidity, and free and total sulfur dioxide concentration were measured according to the methods of the Organisation International de la Vigne et du Vin [12].

#### Instrumental analysis

The sugar, organic acid and alcohol concentrations were assessed using high performance liquid chromatography (HPLC). The frozen samples were centrifuged for 10 minutes, and the supernatant (1 mL) was deproteinized through the addition of 34.5 μL perchloric acid (60% p/v) (Merck). The samples were incubated on ice for 30 min, followed by centrifugation and membrane filtration (0.22 mm pore size; Millipore). A 20-μL sample was used for HPLC injection (Waters 501) with a 2.5 mM solution of H_2_SO_4_ (mobile phase) at a flow rate of 0.6 mL min^-1^. The separation was performed on a size-exclusion and ion-exchange column (8.0 x 300 mm, Shodex SH1011) at 60 °C. The compounds were quantified using a refractive index detector (Waters 2410), and the integration was performed using the chromatography data software Empower-2 (Waters, USA).

### Response Surface Methodology

#### Experimental design

In Response Surface Methodology (RSM), the response *y* is described by a polynomial equation as a function of the *p* independent variables, *x*_*i*_, that is,

$$Y=f\left(x_{1},x_{2},\ldots ,x_{p}\right)+\varepsilon$$

Where *ε* represents the error observed in the response *y*. The response is typically well modeled by a first- or a second-order polynomial representing a (*p*+1) dimensional surface, i.e., the *response surface*. The parameters of these equations are typically unknown and, therefore, must be estimated from the experimental data using the statistical principle of least squares. In second-order equations, the coefficients of the squared terms influence the direction of the curvature of the response surfaces. In the present study, the influence of the three factors (variables) viz., sugar, ethanol and sulfur dioxide, was studied using a central composite rotatable design (CCRD), comprising the following experimental points:

1. A factorial design with 2*p* data points (extremes), representing the vertices of a *p*-dimensional cube, at a distance of *p* ^1/2^ from the origin of the coded system of reference. In the coded matrix, these data points correspond to the levels (−1) and (+1) for each variable (Table 1).
2. A group of 2*p* points on the axes of the system of reference, outside of the factorial matrix, but inside of the experimental domain, at a distance equal to 2^p/4^ from the origin (star-points). These levels correspond to -1.68 and 1.68 (Table 1).
3. A third set comprising the repetition of the points at the origin of the reference system (center points), coded as (0,0).

**Table 1:** Coded matrix of the CCRD experimental design.

Experiment		Variable			Concentrations	
	Glucose	Ethanol	SO_2_	Glucose	Ethanol	SO_2_
				(g L^-1^)	% (v/v)	(mg L-1)
1	-1	-1	-1	3.6	11	8
2	1	-1	-1	8.4	11	8
3	-1	1	-1	3.6	14	8
4	1	1	-1	8.4	14	8
5	-1	-1	1	3.6	11	32
6	1	-1	1	8.4	11	32
7	-1	1	1	3.6	14	32
8	1	1	1	8.4	14	32
9	-1.68	0	0	2	12.5	20
10	1.68	0	0	10	12.5	20
11	0	-1.68	0	6	10	20
12	0	1.68	0	6	15	20
13	0	0	-1.68	6	12.5	0
14	0	0	1.68	6	12.5	40
15 (C)	0	0	0	6	12.5	20
16 (C)	0	0	0	6	12.5	20
17 (C)	0	0	0	6	12.5	20

The 17 CCRD trials were designed to understand the interaction of sulfur dioxide, ethanol and glucose using the following realistic concentrations of each parameter in wines: 0 to 40 mg L^-1^ initial free sulfur dioxide, 10% to 15% (v/v) ethanol and 2 to 10 g L^-1^ glucose (Table 1). These ranges were selected based on previous experiments where both yeast “growth” and “no growth” were observed.

#### Statistical analysis

The results of the 17 trials of each CCRD were analyzed using “Statistica” software version 7 (Statsoft, USA). The linear and quadratic effects were calculated, and the significance of these values was evaluated using analysis of variance. The four-dimensional surface, described by a first- or a second-order polynomial equation, was fitted to each set of experimental data points. First- and second-order coefficients were generated through regression analysis. The fit of the models was evaluated using the determination coefficients (*R* ^2^) and adjusted *R* ^2^ (*R*_adj_^2^) [13].

## Results

The variation in the viability of the test yeasts during incubation in red wine showed 3 different patterns: i) immediate cell death or non-culturabilty after inoculation; ii) death after inoculation, followed by growth recovery; iii) growth or viability maintenance after inoculation (S1 Table). Therefore, the viability values used in RSM were calculated at different incubation times. The results presented herein were obtained after incubation for 2 and 5 days (short period response) or 15 and 30 days (long period response). Because free sulfur dioxide decreased to zero in the wine after incubation for 30 days, longer incubation times were considered unrealistic, as previously demonstrated [9].

### *Schizosaccharomyces pombe*

**Fig 1:**
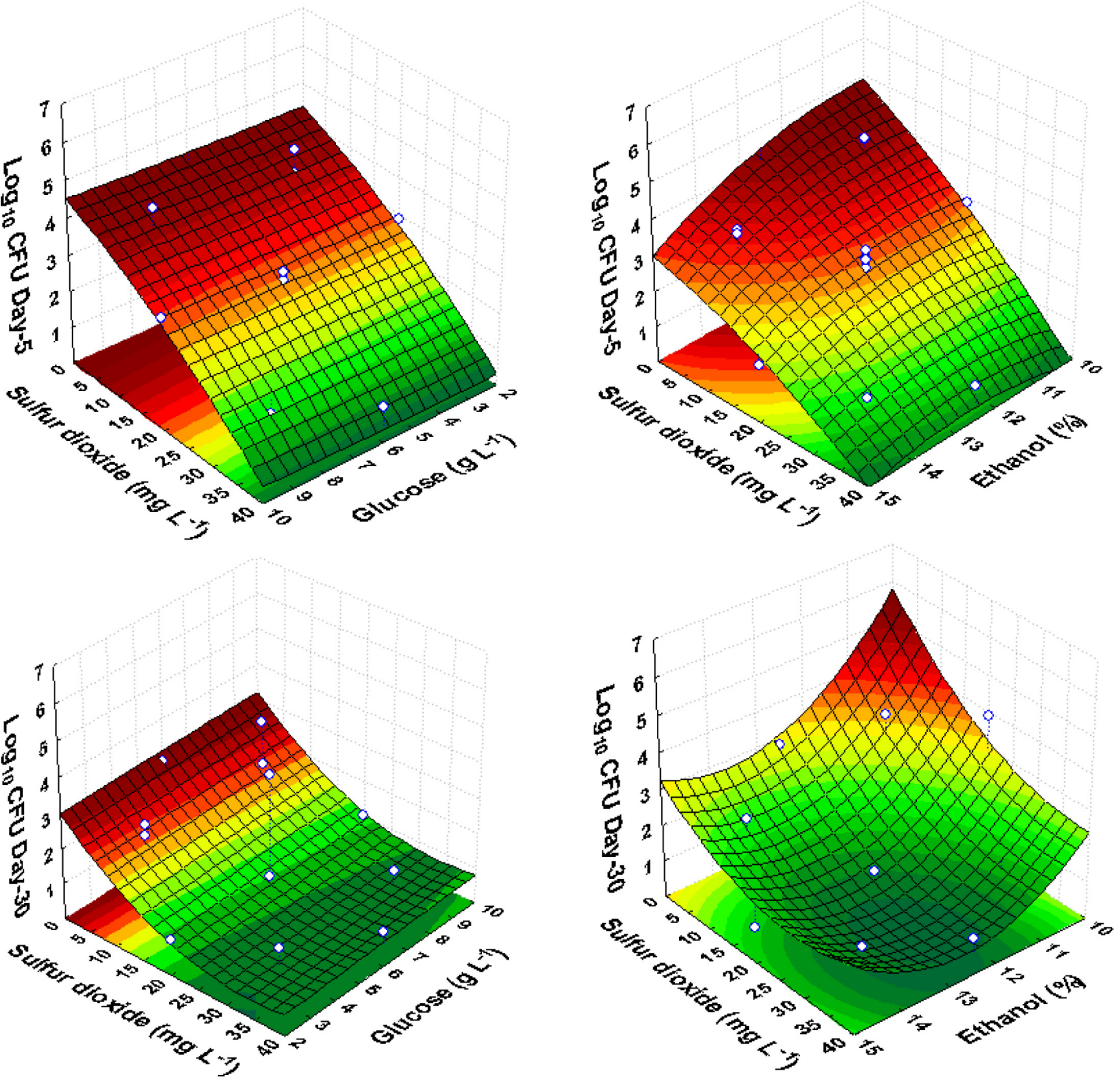
Response surface fitted to experimental data points corresponding to the growth of *Sc*. *pombe* on the 5^th^ and 30^th^ day of observation as a function of glucose, ethanol and sulfur dioxide, in red wine CCRD experiments.

Under high ethanol concentrations, sulfur dioxide showed the highest significant effect (*p*-value < 0.05) on immediate death activation, mathematically confirmed through the negative linear effect of this factor on culturable cells after incubation for 2, 5, 15, and 30 days (Table 2). The response surface plots of the cell growth as a function of sulfur dioxide and ethanol, illustrated the contribution of the negative linear effects of both ethanol and sulfur dioxide to the model, showing that an increase in sulfur dioxide or ethanol promoted a decrease in *Sc*. *pombe* cell numbers. However, the inhibitory effect of ethanol was weaker compared with that of sulfur dioxide. The positive significant quadratic effects of ethanol and sulfur dioxide after incubation for 15 and 30 days, showed that the response surface is concave as a function of these two variables (Fig 1). In general, at a concentration of 32 mg L^-1^ or higher, sulfur dioxide showed immediate death activation. Below this concentration, death activation through sulfur dioxide was only observed in the presence of 12.5% (v/v) or higher ethanol levels (S1 Table).

**Table 2:** Linear and quadratic effects of glucose, sulfur dioxide and ethanol concentrations on the growth of *Sc. pombe*.

Factor				Growth				
	D-2		D-5		D-15		D30	
	Effect	p	Effect	p	Effect	p	Effect	p
Glucose (g/L)(L)	-0.331	0.17993	-1.2	0.079	-0.350	0.575	-0.979	0.456
Glucose (g/L)(Q)	2.384	0.000	2.097	0.014	1.302	0.089	1.603	0.282
(2)Ethanol (%)(L)	-0.339	0.068	-0.598	0.341	-1.277	0.069	-1.928	0.164
Ethanol (%)(Q)	2.529	0.000	2.242	0.011	1.089	0.143	2.250	0.146
(3)Sulfur dioxide (mg/L)(L)	-1.302	0.000	-1.478	0.039	-1.717	0.023	-0.638	0.623
Sulfur dioxide (mg/L)(Q)	1.022	0.001	0.420	0.538	0.223	0.745	0.522	0.715
1 L by 2 L	0.439	0.069	0.621	0.442	0.025	0.975	0.208	0.901
1 L by 3 L	-0.427	0.075	-1.382	0.112	-0.038	0.962	0.024	0.989
2 L by 3 L	-0.178	0.411	-0.269	0.734	1.309	0.135	-0.118	0.944

D-2, D-5, D-15 and D-30 represent observations obtained on 2, 5, 15 and 30 days of incubation, respectively.

Glucose did not support the tolerance of *Sc*. *pombe* cells to ethanol or sulfur dioxide stress. For instance, the comparison of trials 9 and 10 from the CCRD table (Table 1) revealed that viable cells could not be detected in wines containing 2 or 10 g L^-1^ glucose (S1 Table). Table 3 shows the polynomial model equations fitted to the experimental data obtained after incubation for 2, 5, 15 and 30 days. In these equations, only factors with significant effects (p-value <0.05) or those having a confidence range smaller than the value of the effect or smaller than the standard deviation were included. All models had both high R^2^ and adjusted R^2^ values, showing a good fit of these models to the experimental results.

**Table 3:** Model equations describing the response surfaces fitted to the CCRD experimental data points.

Yeast sp.	Parameter	Model equation	R^2^	R^2^-adj
*Shizo*. *pombe*	Log_10_CFU 2 days	47.733–1.706(G)+0.12(G)^2–5.68(Et)+0.20(Et)^2–0.53(S)+0.0006(S)^2+0.0236(G*E)+0.033(S*E)	0.96	0.92
	Log_10_CFU 5 days	-3.200445+0.00164(G)+1.58041(Et)-0.07808(E)^2–0.18864(S)-0.00093(S)^2+0.01(Et*S)	0.91	0.86
	Log_10_CFU 15 days	32.1504+0.00653(G)-4.20252(Et)+0.15851(E)^2–0.29682(S)+0.00385(S)^2+0.00431(Et*S)	0.90	0.84
	Log_10_CFU 30 days	45.3711+0.02776(G)-6.27166(Et)+0.2301(E)^2–0.28049(S)+0.00225(S)^2+0.01045(Et*S)	0.86	0.78
*Z*. *bailii*	Log_10_CFU 2 days	­8.54573+0.52921(G)-0.04199(G)^2+2.1379(Et)-0.09762(Et)^2–0.25787(S)-0,00429(S)^2+0,02651(Et*S)	0.98	0.92
	Log_10_CFU 5 days	1.705+0.92166(G)-0.071(G)^2+0.000722(Et)-0.02(S)-0.00234(S)^2	0.92	0.88
	Log_10_CFU 15 days	44.73229+0.50528(G)-0.03698(G)^2–5.72804(Et)+0.17976(Et)^2–0.51991(S)-0.00065(S)^2+0.03869(Et*S)	0.90	0.82
	Log_10_CFU 30 days	74.9431–0.0009(G)-10.1248(Et)+0.3458(Et)^2–0.6034(S)+0.0012(S)^2+0.0406(Et*S)	0.93	0.89
*S*. *ludwigii*	Log_10_CFU 2 days	58.9201–8.08379(Et)+0.31278(Et)^2–0.29228(S)+0.00326(S)^2)	0.80	0.73
	Log_10_CFU 5 days	­15.342+1.82(G)-0.144(G)^2+3.258(Et)-0.154(Et)^2–0.0619(S)-0.0021(S)^2	0.79	0.67
	Log_10_CFU 15 days	­17.762+0.768(G)+3.4417(Et)-0.151(Et)^2+0.219(S)-0.0452(G*S)	0.52	0.31
	Log_10_CFU 30 days	5.26636–0.00041(G)+0.03045(Et)+0.10624(S)+0.00111(S)^2–0.01041(E*S)	0.53	0.32
*S*. *cerevisiae*	Log_10_CFU 2 days	54.343+0.00196(G)2–7.179(Et)+0.272(Et)2–0.509(S)+0.00682(S)2+0.00586(Et*S)	0.89	0.83
	Log_10_CFU 5 days	92.811–0.0009(G)2–12.766(Et)+0.4707(Et)2–0.661(S)+0.0061(S)2+0.0206(Et*S)	0.79	0.67
	Log_10_CFU 15 days	73.017–1.079(G)-8.902(Et)-0.2809(Et)2–0.6806(S)+0.00504(S) 2+0.0769(G*S)+0.0313(Et*S)	0.52	0.31
	Log_10_CFU 30 days	60.984+0.00877(G)2–8.150(Et)+0.286(Et)2–0.167(S)+0.00325(S)2–0.00815(G*Et)	0.82	0.71

Growth (Log_10_CFU) of test yeasts is represented as a function of the concentration of glucose (G), ethanol (E) and sulfur dioxide (S) and respective R^2^ and R^2^_adj_.

### *Zygosaccharomyces bailii*

**Fig 2:**
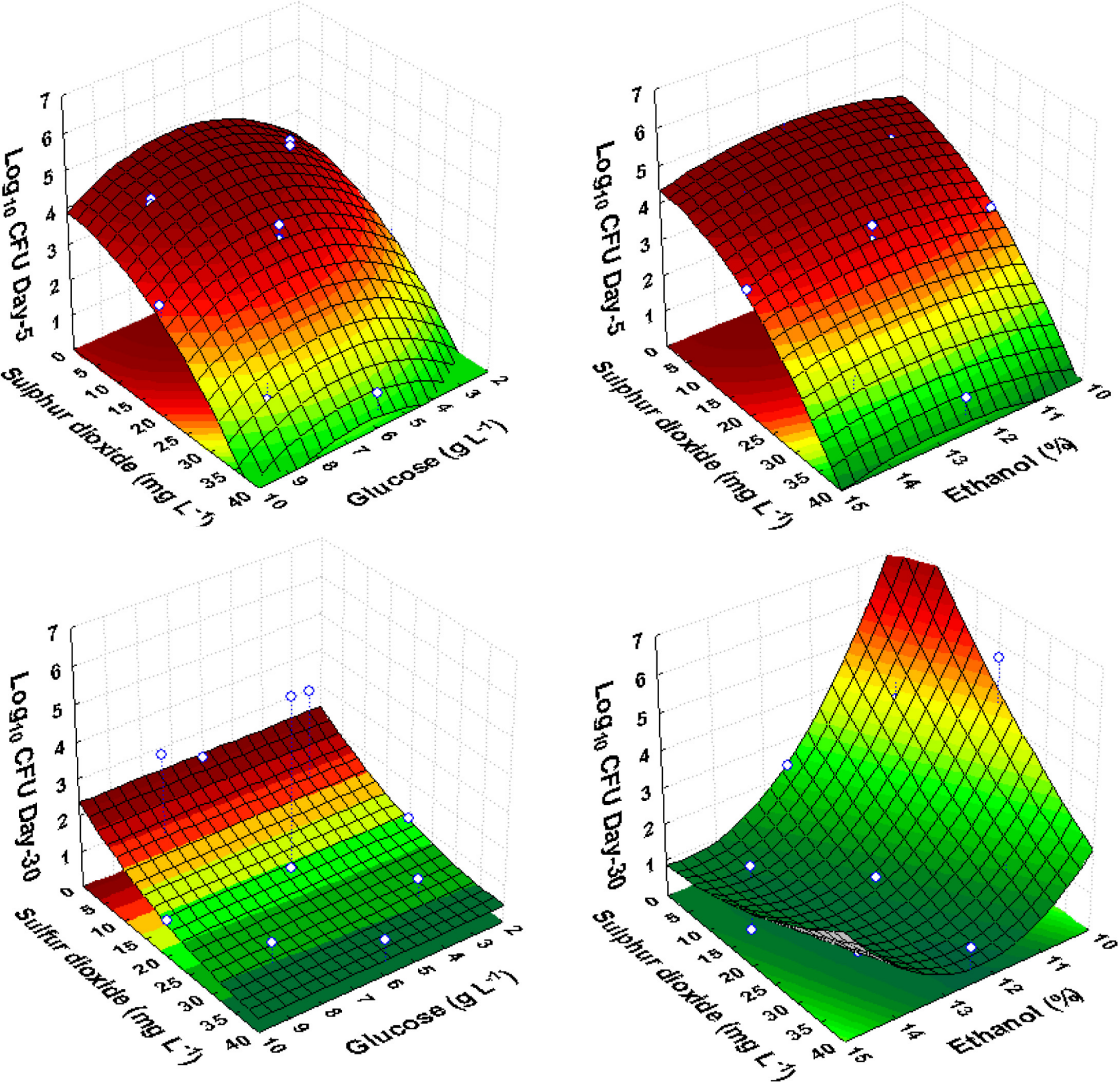
Response surface fitted to experimental data points corresponding to the growth of *Z*. *bailii* on the 5^th^ and 30^th^ day of observation as a function of glucose, ethanol and sulfur dioxide, in red wine CCRD experiments.

The effects (linear, quadratic and interactions) of the factors on *Z*. *bailii* growth after incubation for 30 days as shown in Table 4, revealed the negative linear effect of sulfur dioxide after 2, 5, 15 and 30 days. In most of the CCRD trials, yeast populations slowly decreased with time when the sulfur dioxide level was 20 mg L^-1^ or higher in presence of 12.5% (v/v) ethanol. However, cells exposed to 32 mg L^-1^ or higher sulfur dioxide concentrations showed immediate death that was confirmed by non-culturability on surface media. Moreover, similarly to *Sc*. *pombe*, *Z*. *bailii* cells exposed to this level of sulfur dioxide could never regain growth. The response surface was fitted to the experimental results (Table 3), showing a convex shape as a function of sulfur dioxide levels (Fig 2). With respect to ethanol concentration, a negative quadratic effect (convex surface) was observed after incubation for 2 and 5 days, but after 15 and 30 days, a positive quadratic effect was observed, suggesting concave response surfaces.

**Table 4:** Linear and quadratic effects of glucose, sulfur dioxide and ethanol concentrations on the growth of *Z. bailii*.

Factor				Growth				
	D-2		D-5		D-15		D30	
	Effect	p	Effect	p	Effect	p	Effect	p
Glucose (g/L)(L)	0.121	0.463	0.335	0.298	0.296	0.377	0.296	0.377
Glucose (g/L)(Q)	-0.484	0.027	-0.923	0.027	-0.426	0.260	-0.426	0.260
(2)Ethanol (%)(L)	0.683	0.003	0.072	0.816	-1.380	0.003	-1.380	0.003
Ethanol (%)(Q)	-0.439	0.039	-0.364	0.306	0.809	0.053	0.809	0.053
(3)Sulfur dioxide (mg/L)(L)	-2.350	0.000	-2.729	0.000	-1.497	0.002	-1.497	0.002
Sulfur dioxide (mg/L)(Q)	-1.234	0.000	-0.780	0.050	-0.188	0.606	-0.188	0.606
1 L by 2 L	0.039	0.855	-0.005	0.990	0.066	0.875	0.066	0.875
1 L by 3 L	0.056	0.792	-0.019	0.962	-0.084	0.842	-0.084	0.842
2 L by 3 L	0.954	0.002	0.077	0.849	1.393	0.011	1.393	0.011

D-2, D-5, D-15 and D-30 represent observations obtained on 2, 5, 15 and 30 days of incubation, respectively.

*Z*. *bailii* growth was accompanied by partial glucose consumption (data not shown). Glucose showed a positive linear effect on culturable cells (Table 4). However, this positive effect could not counteract the toxic effects of high levels of sulfur dioxide and ethanol. The short-term response of *Z*. *bailii* was described by a convex surface as a function of glucose concentration, indicating that glucose presented a significant quadratic effect (Fig 2). However, the long-term response indicated that effect of sugar was no longer significant.

### *Saccharomycodes ludwigii*

**Fig 3:**
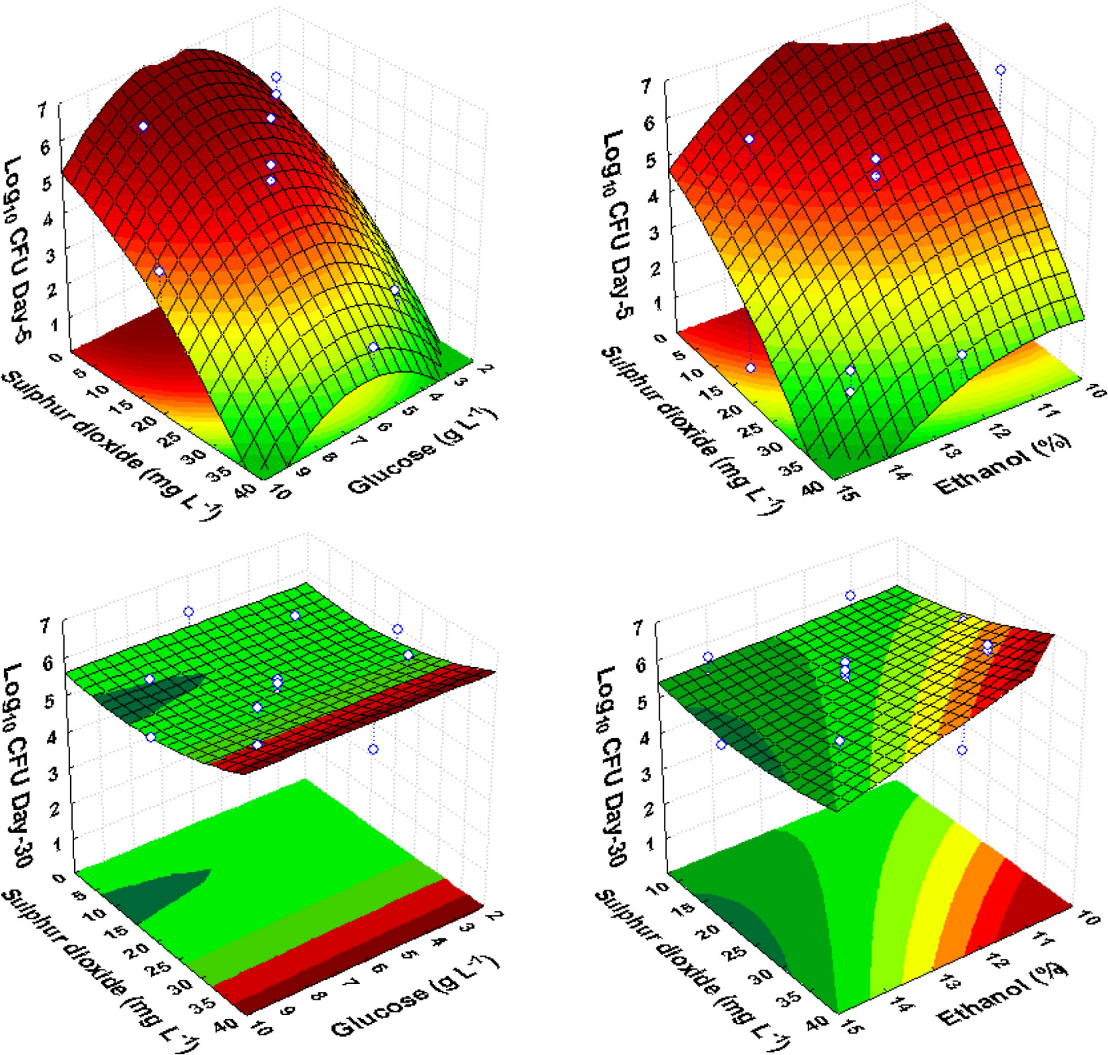
Response surface fitted to experimental data points corresponding to the growth of *Sa*. *ludwigii* on the 5^th^ and 30^th^ day of observation as a function of glucose, ethanol and sulfur dioxide, in red wine CCRD experiments.

For *Sa*.*ludwigii*, ethanol and sulfur dioxide concentrations showed significant negative linear effects on yeast growth (Table 5). Glucose supported the growth of yeast in short-term incubations (2 and 5 days), evidenced as a positive linear effect, but not in long-term incubations (15 and 30 days) (Table 5). The response surface, as a function of sulfur dioxide and ethanol, showed the significant contribution of sulfur dioxide to the model, confirmed by a negative quadratic effect until day 15 (Fig 3, Table 5). However, the negative linear effect of sulfur dioxide disappeared by the 30^th^ day of incubation. In some CCRD trials, growth recovery was not identical under higher sulfur levels (32 mg L^-1^), showing growth recovery on the 5^th^ day in some cases and on the 30^th^ day in other cases. We observed that trials with higher sulfur dioxide concentrations (32 mg L^-1^) in the presence of increased levels of glucose required more time to regain viability, suggesting that glucose did not stimulate the recovery of viability. The response surface was fitted to the experimental points, showing that this yeast maintained growth, even at the highest test concentrations of ethanol and sulfur dioxide (Fig 3). This result indicates the high resistance of *Sa*. *ludwigii* to these inhibitors. Indeed, even after 30 days of growth, the population of *Sa*. *ludwigii* was consistently higher than 5 log_10_ CFU mL^-1^ under all conditions tested (S1 Table).

**Table 5:** Linear and quadratic effects of glucose, sulfur dioxide and ethanol concentrations on the growth of *Sa. ludwigii*.

Factor				Growth				
	D-2		D-5		D-15		D30	
	Effect	p	Effect	p	Effect	p	Effect	p
Glucose (g/L)(L)	0.492	0.389	0.431	0.609	-0.646	0.517	-0.081	0.767
Glucose (g/L)(Q)	-1.241	0.075	-1.662	0.105	-0.609	0.581	0.341	0.280
(2)Ethanol (%)(L)	-0.793	0.182	-1.777	0.063	-1.025	0.315	-0.533	0.082
Ethanol (%)(Q)	0.288	0.642	-0.693	0.463	-0.859	0.441	0.080	0.792
(3)Sulfur dioxide (mg/L)(L)	-3.884	0.000	-3.523	0.003	-1.227	0.237	0.495	0.101
Sulfur dioxide (mg/L)(Q)	-0.180	0.770	-0.611	0.515	-0.191	0.861	0.415	0.197
1 L by 2 L	-0.136	0.850	-0.192	0.860	0.458	0.721	0.272	0.452
1 L by 3 L	0.043	0.953	-0.269	0.804	-2.602	0.073	0.086	0.808
2 L by 3 L	0.615	0.406	0.424	0.697	-0.206	0.872	-0.375	0.309

D-2, D-5, D-15 and D-30 represent observations obtained on 2, 5, 15 and 30 days of incubation, respectively.

### *Saccharomyces cerevisiae*

**Fig 4:**
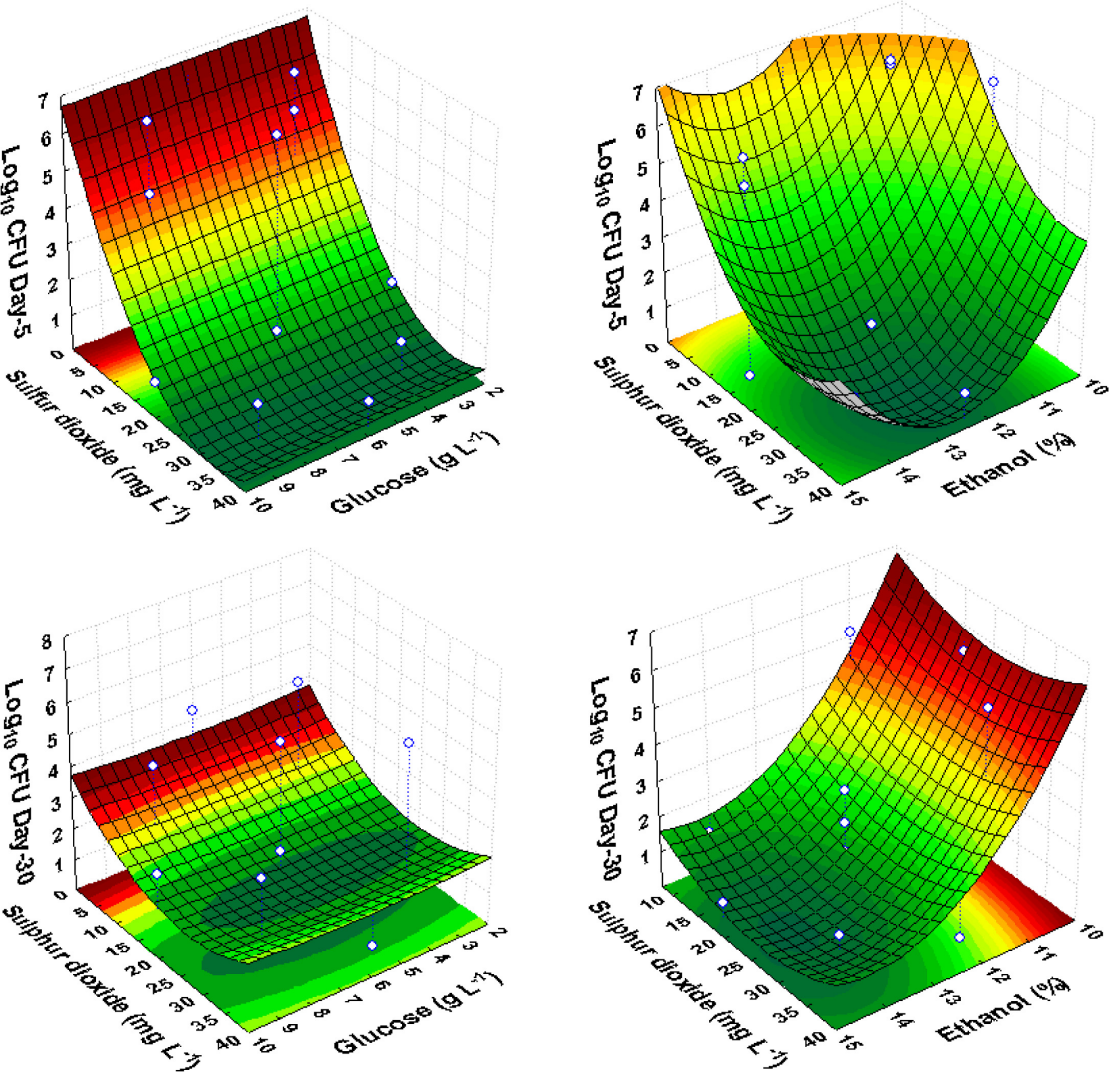
Response surface fitted to experimental data points corresponding to the growth of *S*. *cerevisiae* on the 5^th^ and 30^th^ day of observation as a function of glucose, ethanol and sulfur dioxide, in red wine CCRD experiments.

Similar to the other test yeasts, responses under high ethanol and sulfur dioxide conditions, showed that sulfur dioxide had the most significant effect (p-value <0.05) on *S*. *cerevisiae* growth (Table 6). Immediate death activation was observed with 32 and 40 mg L^-1^ of sulfur dioxide. However, at sulfur dioxide concentrations less than 32 mg L^-1^ in the presence of low levels of ethanol (11% v/v), these cells could regain growth after some period of time. The effect of glucose on *S*. *cerevisiae* was similar to *Sc*. *pombe* and *Z*. *bailii*, as revealed by the shape of response surfaces. (Fig 4).

**Table 6:** Linear and quadratic effects of glucose, sulfur dioxide and ethanol concentrations on the growth of *S*. *cerevisiae*.

Factor				Growth				
	D-2		D-5		D-15		D30	
	Effect	p	Effect	p	Effect	p	Effect	p
Glucose (g/L)(L)	0.022	0.971	-0.096	0.884	-0.563	0.200	-0.045	0.937
Glucose (g/L)(Q)	0.584	0.395	0.270	0.714	0.169	0.712	0.472	0.458
(2)Ethanol (%)(L)	-0.747	0.239	-1.756	0.028	-2.371	0.001	-3.126	0.001
Ethanol (%)(Q)	1.387	0.068	2.198	0.017	1.313	0.021	1.395	0.053
(3)Sulfur dioxide (mg/L)(L)	-3.914	0.000	-3.822	0.001	-2.106	0.001	-0.894	0.143
Sulfur dioxide (mg/L)(Q)	2.124	0.013	1.837	0.035	1.501	0.011	1.041	0.127
1 L by 2 L	0.072	0.926	-0.224	0.795	0.554	0.320	0.792	0.298
1 L by 3 L	-0.162	0.836	0.163	0.850	-0.112	0.835	-0.350	0.634
2 L by 3 L	0.211	0.788	0.743	0.400	1.127	0.066	0.139	0.849

D-2, D-5, D-15 and D-30 represent observations obtained on 2, 5, 15 and 30 days of incubation, respectively.

Overall, sulfur dioxide was demonstrated as an inhibitory factor at higher concentrations, confirmed by the negative linear effect of this factor on culturable cell populations observed at all time intervals (Table 6). Moreover, the dominant contribution of the positive quadratic effect of sulfur dioxide to this model could be clearly observed in concave response surface plots of the cell growth as a function of sulfur dioxide and ethanol (Fig 4). In the presence of low levels (8 mg L^-1^) of sulfur dioxide and ethanol concentrations up to 12.5% (v/v), *S*. *cerevisiae* cells did not show any inhibitory effect. The relatively stronger toxic effect of ethanol compared with sulfur dioxide was observed by the surface shape after incubation for 30 days, indicating that sulfite was no longer inhibitory (Fig 4).

## Discussion

Using RSM approach, we described the interactions of sulfur dioxide, ethanol and sugar during cellular growth and death. Although all the four tested yeast species showed quantitatively different susceptibilities, we observed a similar overall response characterized by i) growth inhibition due to the effect of sulfur dioxide and ethanol; ii) viability recovery when stress is alleviated; and iii) the absence of growth stimulation by glucose under ethanol and sulfite stresses. Response surfaces and model equations of CCRD trials reflected both sulfur dioxide and ethanol as inhibitory agents. The inhibitory effect of ethanol was primarily observed during longer incubation periods, contrary to sulfur dioxide, which showed an immediate effect. Consistently, Sturm et al. [14] reported that the combined inhibition of ethanol and sulfur dioxide was more efficient during short-term incubation periods, rather than long-term incubation periods. This result might reflect the loss of free sulfite during longer incubation periods [9]. The loss of sulfite might also contribute to the recovery of viability under growth-permitting ethanol concentrations.

Apart from the wine compositions, container geometry and evaporation, sulfur dioxide combination with phenolic compounds is one of the major reasons of sulfite loss in wine. Based on winery level studies, barrels showed sulfur dioxide levels reduction within a period of four to six months of ageing [15], and therefore, sulfur dioxide management is crucial during this time. As a result, opposed to larger and less frequent additions, frequent small sulfur dioxide additions should be performed. However, this practice may not be helpful to solve the spoilage problem, as frequent small sulfur dioxide additions can lead to the unintentional selection or build-up of more resistant yeast species [16].

Given that fermentable sugar is the primary carbon and energy source for yeasts, it was unexpected to observe that glucose did not prevent cellular death under sulfur dioxide and ethanol stress in most cases. In fact, glucose up to 10 g L^-1^ did not increase the wine susceptibility to the growth of *S*. *cerevisiae*, *Sc*. *pombe* and *Z*. *bailii*. In case of *Sa*. *ludwigii*, the absence of cell death under the highest concentrations of SO_2_ and ethanol did not allow to evidence this behavior. We assume that growth recovery in the case of *Sa*. *ludwigii* did not occur as a function of higher glucose levels.

The occurrence of growth recovery indicates the potential existence of viable but non-culturable (VBNC) cells, a state of cell majorly studied in bacteria. This state is characterized by an inability of the cells to grow on culture media, while maintaining their detectable metabolic activity [17]. Various environmental factors such as temperature [18, 19], the physiological age of the culture, salinity [20], the oxygen content [20], light and ventilation [21] are known to induce VBNC state in bacteria. Recent studies have indicated that sulfur dioxide induces a VBNC state in the wine spoilage yeast *Brettanomyces* [22, 23]. *Candida stellata*, *S*. *cerevisiae* and *Z*. *bailii* have also been shown to have VBNC state during alcoholic fermentation of wines [24, 25]

In the case of VBNC, when stress is alleviated, the cells regain culturability [26], as recently shown for *B*. *bruxellensis* [26–29] and *S*. *cerevisiae* [30]. To the best of our knowledge, this recovery has not been demonstrated for *Sa*. *ludwigii*. As these species are highly tolerant to stresses [31], it would be interesting to determine whether this feature is associated with the ability of yeast cells to enter the VBNC state and subsequently recover when the stresses are alleviated.

## Conclusion

The inhibitory effect of ethanol and sulfur dioxide is consistent with scientific and empirical knowledge. Growth recovery of *S*. *cerevisiae* and *Sa*. *ludwigii* indicates possible existence of VBNC state. Though VBNC state in *S*. *cerevisiae* has already been reported, such behavior in *Sa*. *ludwigii* is reported for the first time to the best of our knowledge. However, further studies are required to confirm the existence of VBNC state in this yeast species under the exposure of sulfite and other stressors. The absence of an evident glucose effect on cell growth was unexpected. Lightly sweetened table wines (with a soften mouthfeel), are thought to be more prone to yeast spoilage [32]. However, the results obtained in the present study did not support this view. The synergistic effect of ethanol and sugar on growth inhibition has been previously demonstrated in fortified wines at much higher levels than examined in the present study [32]. Moreover, the results of present study are consistent with the recent findings concerning *B*. *bruxellensis* [9], where we observed the absence of glucose effect on the prevention of cell death. We hypothesized that under growth permeable conditions, increased levels of glucose support higher biomass production, but under ethanol and/or sulfur dioxide stresses, high glucose concentrations do not facilitate stress recovery.

The species studied herein are regarded as the most dangerous wine spoilage agents, as these yeasts are highly tolerant to the stresses imposed in wine [33–35]. Our results are important to wine industry because they show that wines sweetened with sugar do not require higher doses of preservatives to achieve microbial stability, as commonly believed. In the present study, *Sa*. *ludwigii* was recorded to be the most resistant species. This yeast species has already been mentioned as the “winemaker’s nightmare” by Thomas [34]. *Sc*. *pombe* showed similar resistance to *S*. *cerevisiae* and slightly higher resistance than *Z*. *bailii*. However, *Sa*. *ludwigii* and *Sc*. *pombe* are much less frequently isolated from spoiled wines than *Z*. *bailli*, *S*. *cerevisiae* or *B*. *bruxellensis* [32, 33, 35]. *Sa*. *ludwigii* Therefore, the reasons for the higher overall incidence of the latter species in wines might be associated with physiological features other than tolerance to ethanol or sulfur dioxide stress.

## Supporting Information

S1 Table Growth of *Sc*. *pombe*, *Z*. *bailii*, *S*. *lugwigii* and *S*. *cerevisiae* in the test wine as a function of glucose, ethanol and sulfur dioxide concentrations. (DOCX)
