# Supplementary material for: Effect of Ethanol, Sulfur Dioxide and Glucose on the Growth of Wine Spoilage Yeasts Using Response Surface Methodology
Source: PLoS One. 2015 Jun 24;10(6):e0128702. doi: 10.1371/journal.pone.0128702 (PMC4479441; doi:10.1371/journal.pone.0128702)
Supplement: S1 Table — (DOCX) [file pone.0128702.s001.docx]

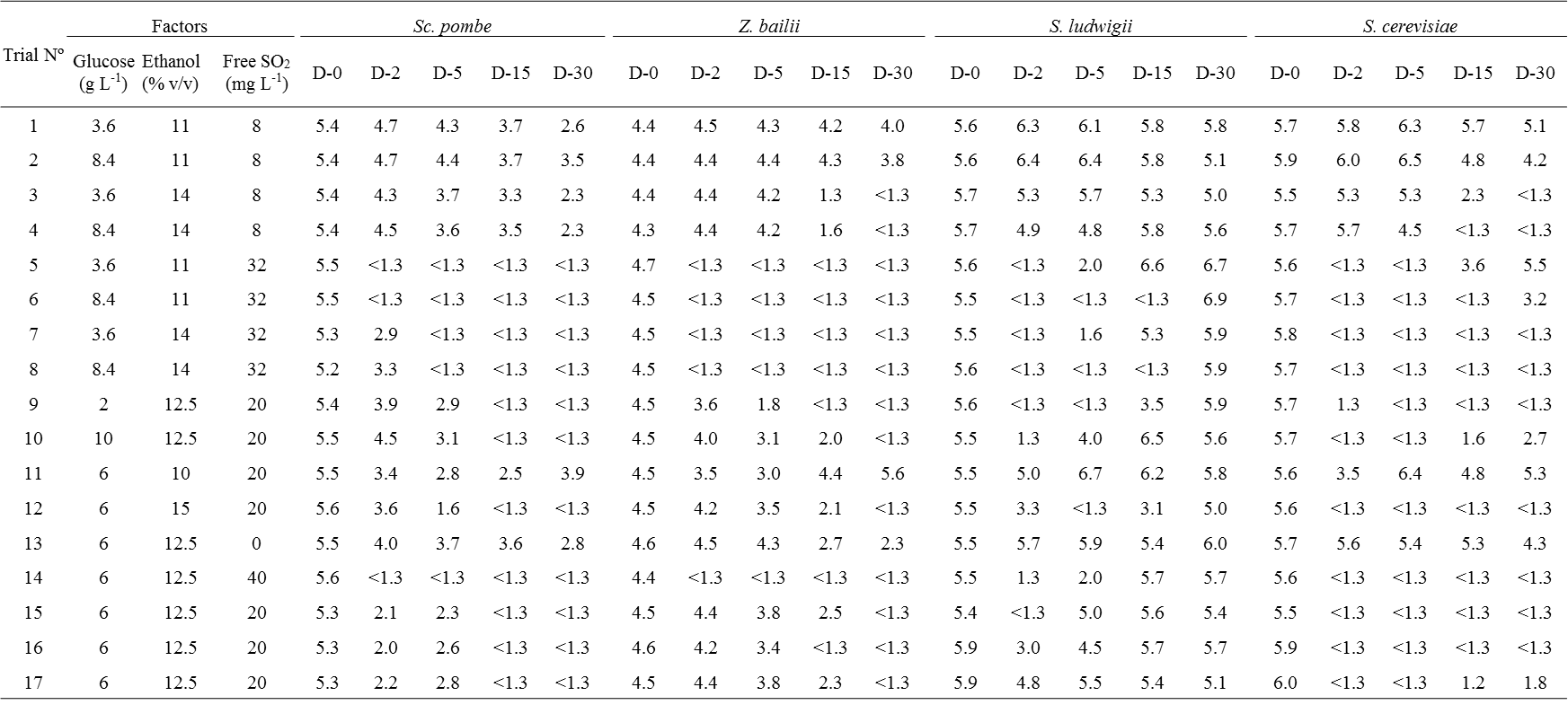
S1 Table. Growth of *Sc. pombe*, *Z. bailii*, *S. lugwigii* and *S. cerevisiae* in the test wine as a function of glucose, ethanol and sulphur dioxide concentrations.

D-0, D-2, D-5, D-15 and D-30 represent observations taken on 0, 2, 5, 15 and 30 days of incubation, respectively.
